# Analysis of risk factors related to gastrointestinal fistula in patients with severe acute pancreatitis: a retrospective study of 344 cases in a single Chinese center

**DOI:** 10.1186/s12876-017-0587-8

**Published:** 2017-02-14

**Authors:** Zhipeng Hua, Yongjie Su, Xuefeng Huang, Kang Zhang, Zhengyu Yin, Xiaoming Wang, Pingguo Liu

**Affiliations:** 1 Department of Hepatobiliary Surgery, Zhongshan Hospital of Xiamen University, Hubing South Road, Xiamen, Fujian China; 2 Fujian Provincial Key Laboratory of Chronic Liver Disease and Hepatocellular Carcinoma (Xiamen University Affiliated ZhongShan Hospital), Xiamen, China

**Keywords:** Severe acute pancreatitis, Gastrointestinal fistula, Risk factor, Infected pancreatic necrosis, MCTSI, EEN, Blood type B

## Abstract

**Background:**

Gastrointestinal fistula (GIF) in severe acute pancreatitis (SAP) is considered as a sparse episode and studied sporadically in the literature. There is paucity of data on the prediction of the effect on risk of GIF in patient with SAP. This study was aimed to investigate risk factors related to GIF in the development of SAP.

**Methods:**

The clinical data of 344 patients with SAP from 2011 to 2016 were reviewed retrospectively. All patients were divided into the GIF group and the non-GIF group, and their data analyzed with respect to 15 parameters were applied to explore potential risk factors for GIF in patients with SAP.

**Results:**

Of the 344 eligible patients, 52 (15.12%) progressed to GIF. Only occurrence of infected pancreatic and extra-pancreatic necrosis (IPN) (*P* = 0.004, OR = 3.012) and modified CT severity index (MCTSI) (*P* = 0.033, OR = 1.183) were proved to be independent risk factors for GIF in patients with SAP, and blood type B (*P* = 0.048, OR = 2.096, 95% CI: 0.748–3.562) indicated weaker association of risk factor for GIF. The early (48–72 h after admission) enteral nutrition (EEN) (*P* = 0.016, OR = 0.267) acted as a protective factor.

**Conclusions:**

Occurrence of IPN and high MCTSI are independent risk factors for the development of GIF in patients with SAP, blood type B reveals a potential correlation with GIF in patients with SAP. EEN is helpful to prevent the progression of GIF secondary to SAP.

## Background

Severe acute pancreatitis (SAP) is a devastating disease that is characterized by a high mortality rate (ranging from 15% to as high as 85%) due to the development of pancreatic and extra-pancreatic necrosis infection, and multi-system organ failure (MOF) [1, 2]. The management of SAP is complicated because of the incomplete understanding of the pathogenesis and multi-causation of the disease, uncertainties in predicting outcome and limited effective treatment modalities [2, 3]. Gastrointestinal fistula (GIF) is a well-recognized complication secondary to SAP, although the incidence of GIF in SAP is low and sporadically reported in the literature. As previously reported, GIF is one of the most fatal and intractable complications after SAP, and associated with other major complications and serious clinical consequences, such as hemorrhage and exacerbation of infection which can lead to a fatal outcome [4–7]. The etiology and pathogenesis of GIF in patients with SAP involve complex processes, which are far from fully understood. Indeed, the management of GIF in SAP is complicated and controversial, which could lead to a prolonged hospital course, and significant morbidity and mortality [8, 9]. The sites of fistula may involve the stomach, duodenum, jejunum, ileum, and colon, either in localization or diffusion. GIF may result from direct erosion from digestive enzymes excreted by the inflamed pancreas on the adjacent gastrointestinal (GI) tract, or it could occur as a consequence of intestinal necrosis due to vascular thrombosis in an area of inflammation and infection. In addition, GIF may be associated with iatrogenic intervention [10–12].

It has been reported that GIF may cause none of additional symptoms in some cases, which are usually detected incidentally on radiologic imaging or during surgical intervention [10, 13–15]. The resulting events of GIF we observed also confused us frequently, which led to either further complications or spontaneous resolution. Interestingly, more of GIF often tended to relatively facile resolution rather than thorny complications, especially serious GIF, such as the case of multiple or diffuse. Little data exists regarding the risk factors for this complication, and few publications provide precise and adequate predictions of the risk for GIF in patients with SAP. Therefore, the early prediction of GIF and specific targeted interventions are imperative to reduce GIF-related mortality [16, 17]. In this retrospective study we analyzed the data from patients with SAP to determine the risk factors for developing GIF. Moreover, we also studied the different clinical characteristics and outcomes of GIF in the setting of SAP.

## Methods

### Patient enrollment

From January 2011 to January 2016, patients with a primary diagnosis of SAP admitted to Departments of Emergency, Hepatopancreatobiliary Surgery, Gastroenterology, Surgical Intensive Care Units of Zhongshan Hospital (Xiamen, China) within 72 h from the onset of the disease were screened for enrollment, and including some critical patients confirmed SAP who transferred from other facilities. Demographic and clinical characteristics of patients were collected at the time of admission.

Our criteria are consistent with that recommended in the Revised Atlanta Classification (RAC-2013) [18] and the revised guidelines of the Italian Association for the Study of the Pancreas (AISP-2014) [9]. To ensure the inclusion of only eligible patients with SAP, only those with an acute inflammatory process of the pancreas associated with variable severity were included, such as the presence of organ failure and local/systemic complications. Patients who met the following criteria were excluded: (1) patients developed GIF after iatrogenic intervention or surgical management;(2) younger than 18 years old age; (3) previous diagnosis of chronic liver and gastrointestinal disease; (4) pregnancy or severe immune system disorders; (5) end-stage chronic disease; (6) patients with incomplete data (e.g., deceased within 24 h after admission, missing computed tomography (CT) diagnosis, or termination of treatment on halfway); (7) patients with chronic pancreatitis, known malignancy were excluded.

### Diagnosis and classification of SAP

According to RAC-2013 and AISP-2014, the diagnosis of SAP requires clinical course, laboratory parameters and imaging evaluation such as contrast-enhanced CT (CECT), ultrasonography (US) and/or magnetic resonance imaging (MRI), Endoscopic ultrasound (EUS) [19–21]. The severity of SAP is stratified moderately severe (MSAP) and severe (SAP). MSAP is defined as the presence of transient organ failure (<48 h), local complications or exacerbation of comorbid disease. SAP is defined as persistent organ failure (> 48 h) affecting respiration, renal function or the cardiovascular system. The SAP diagnosis requires at least one of the following criteria: (a) Acute Physiology and Chronic Health Evaluation II (APACHE II) score 8; (b) Ranson score 3; (c) organ failure (i.e., transient and persistent); and (d) local complications (i.e., necrosis, abscess or pseudocyst) [22]. The presence of organ failure was defined by Modified Marshall Scoring System [18, 23]. Local complications have been defined in RAC-2013, include acute pancreatic or peripancreatic fluid collection (APFC), acute necrotic collection (ANC), and walled-off necrosis (WON), pancreatic pseudocysts. IPN is defined as the presence of infection in the development of ANCs and WONs. Other local complications include pancreatic fistula, gastric outlet dysfunction, splenic and portal vein thrombosis, gastrointestinal necrosis and fistula, hemorrhage etc [24–26]. Systemic complications are involved as exacerbation of preexisting conditions like systemic inflammatory response syndrome (SIRS), coronary artery disease, congestive cardiac failure, chronic obstructive pulmonary disease, diabetes, and chronic liver disease, precipitated by acute pancreatitis [27]. GIF is defined as pathological communications that connect any portion of GI tract with the necrotic cavity, the peritoneal space, the retroperitoneal areas, or another internal organ. For overlapped with clinical manifestations of pancreatitis, diagnosis of GIF is often based on fistulography, digestive endoscopy, or operative findings [28].

### Etiologies

The etiology was considered to be of biliary origin when biliary tract stones were detected by US, CT or magnetic resonance cholangiopancreatography (MRCP), endoscopic retrograde cholangiopancreatography (ERCP). Alcohol was considered to be secondly etiological factor. For pancreatitis due to hypertriglyceridemia (HTG), a serum triglyceride (TG) level of more than 1000 mg/dL or 500–1000 mg/dL with a history of HTG was necessary, in addition to exclusion of other triggers. Lacking any of the above evidence or other direct causes, any unexplained pancreatitis, such as sphincter of Oddi dysfunction, pregnancy associated, ampullary obstruction, hyper-calcemia, drugs related and autoimmune, were defined as idiopathic AP in this study [29].

### Clinical management protocol

Immediately after admission, all patients administrated individualized conservative therapy for SAP that included intensive monitoring, fluid resuscitation, oxygen administration, fasting, analgesia and suppression of pancreatic exocrine function by pharmacological agents, such as somatostatin. Nasoduodenal feeding tubes were placed, and feeding was initiated 48^_^120 h after admission. EEN was defined as feeding within 48^_^72 h after admission. Additionally, antibiotic therapies were guided by the results of culture and sensitivity. Rather than preventing infection, antibiotics were prescribed in these often critical patients with established infected necrosis or the presence of other infections (e.g., biliary tract, urinary tract, pulmonary, etc.). CECT was performed routinely for all patients within 72 h after admission or earlier when warranted by diagnostic dilemmas. IPN was diagnosed according to the positive gram stain and culture results of pancreatic or peripancreatitic necrotic tissue obtained by means of CT guided fine needle aspiration, or from the first percutaneous drainage or operation. IPN, WON and pseudocyst with complications were managed with a minimally invasive based step-up approach firstly, next step was performed if there was no clinical improvement. Nonsurgical procedures included percutaneous drainage and continuous negative pressure irrigation. Once GIF in patients with SAP was confirmed, which in most cases were already at least 2 weeks after initiating EEN. Enteral nutrition (EN) was deprived conditionally, and intra or extra-luminal drainage (applicable for the localized GIF), or enterostomy (applicable for the diffuse GIF) might be performed if necessary by the rationale of minimally invasive procedure, as well as interventional management with a step-up approach for SAP.

### Data collection

Data pertaining to clinical characteristics, including laboratory parameters, imaging record, phase and location of GIF, intervention for GIF, and outcomes were recorded. The metrics analyzed in the present investigation included demographic characteristics like age, gender, cause of illness, and clinical parameters such as MCTSI, APACHE II score, C-reactive protein (CRP) level, intra-abdominal pressure (IAP), blood type and occurrence of IPN. All the laboratory results were obtained at the Central Laboratory of Zhongshan Hospital according to the standard protocols. IAP was measured with a catheter inserted into the bladder, and patients underwent EEN were also documented.

### Statistical analysis

SPSS 22.0 software (IBM SPSS Statistics; IBM Corporation; Armonk, NY) was used for data analysis. The distributions of quantitative variables were tested. Normally and non-normally distributed quantitative variables were presented as the median (interquartile range), respectively. Continuous variables were compared between the groups using an unpaired *t*-test and a paired *t*-test within each group. Categorical variables were compared using the Chi-square test. For small samples, analysis of variance and Fisher’s exact test were used to analyze continuous and categorical variables as appropriate. Statistical significance was set at *P* < 0.05.

To identify risk factors for GIF, several series of univariate logistics regression analyses were performed involving 15 indices above mentioned. Variables that showed statistical significance were tested in further multiple logistic regression analyses with the stepwise method.

## Results

During the observational period, 344 patients were enrolled in the analyses. The demographic data and clinical characteristics of both GIF and non-GIF groups are shown in Table 1. Of the 344 patients, GIF developed in 52 patients (15.12%) and most of the GIF cases were confirmed clinically 4-8 weeks after onset of the disease. Table 2 shows the results of the univariate regression analysis of GIF in SAP. Hyperlipidemia, MCTSI, APACHE II score, EEN, B blood type and WON showed significant difference between patients with or without GIF. The results of our study correlate well with the statistical results shown in Table 1, which also suggests differences in respect of these six parameters between GIF and non-GIF patients. Taking these significant variables by univariate analysis together into the multiple logistic regression model as showing in Table 3, Only occurrence of IPN (*P* = 0.004, OR = 3.012) and MCTSI (*P* = 0.033, OR = 1.183) were proved to be independent risk factors for GIF in SAP. EEN (*P* = 0.016, OR = 0.267) confirmed as a protective factor for GIF in patients with SAP. Unfortunately, blood type B (*P* = 0.048), although just marginal statistical significance was reached, but the 95% confidence interval (0.748–3.562) observed in the multivariate logistic regression are paradoxical. Table 4 demonstrates the general characteristics and outcome data of SAP with GIF. Most of GIF (92.3%) occurred beyond the phase of APFC, and diffuse GIF was rarely found in WON. All localized GIF were managed using non-surgical procedures. Forty of 52 fistulas closed spontaneously over time after drainage and the source of infection was controlled. Eight of 40 fistulas closed spontaneously with nothing but conservative supportive management. Seven of 40 patients (17.5%) failed to survive due to MOF or septic shock. For 10 of 12 diffuse GIF, ileostomy or colostomy was performed. Two of them were managed by percutaneous drainage procedure because the patients could not tolerate surgery. Five of fistulas survived and seven (58.3%) died of MOF or other serious complications. The overall mortality was 14 of 52 (26.9%).

**Table 1:** Demographic data and clinical characteristics of the patients with SAP

Characteristic	GIF (n =52)	Non-GIF(n =292)	Total(n = 344)	P value
Age, years(range)	51 (34–77)	49(27–70)	50(27–77)	0.572
Gender, M/F	32/20	160/132	192/152	0.047
Etiology				
Biliary	29	156	185	
Alcohol	7	35	42	
Hyperlipidemia	5	37	42	
Idiopathic	11	64	75	
BMI (kg/m2)	22.84(16.25–25.72)	24.13(16.93–28.32)	23.05(16.20–28.42)	0.053
EEN	10	204	214	0.001
APACHE II score	16(11–19)	10(8–15)	10(8–16)	0.035
MCTSI	8(6–10)	6(6–8)	6(6–10)	0.039
CRP level(mg/dl)	143.0(85.0–186.0)	124.6(50.6–184.3)	128.0(64.5–187.0)	0.027
Albumin	10(8–20)	14(11–24)	13(10–28)	0.046
B blood type	16	55	71	0.022
IAP(mmHg)	10.50(9–12.48)	8.50(6.35–11.50)	9.20(6.54–11.75)	0.093
ascites	30	152	182	0.419
thrombosis	9	38	47	0.181
IPN	43	137	180	0.032
death	14	62	75	0.754

*M* male, *F* female, *APACHE* Acute Physiology and Chronic Health Evaluation, *BMI* body mass index, *EEN* the early enteral nutrition, *CRP* C-reactive protein, *IAP* intra-abdominal pressure, *MCTSI* modified CT severity index, *IPN* infected pancreatic and extra-pancreatic necrosis

**Table 2:** Univariate logistic regression analysis of GIF

Variable	OR	95% CI		*P* value
		Lower	Upper	
Age	1.406	0.972	1.732	0.937
Gender	1.031	0.948	1.431	0.873
Alcohol	1.370	0.253	0.724	0.284
Hyperlipidemia	2.471	0.542	2.797	0.029
BMI	1.151	1.017	1.314	0.056
APACHE II score	1.632	0.951	3.118	0.044
MCTSI	4.233	1.026	4.965	0.025
CRP level	1.973	0.927	2.531	0.172
EEN	0.346	0.253	0.764	0.004
Albumin	2.427	0.862	2.253	0.122
B blood type	2.994	1.181	6.137	0.036
IAP	1.038	0.929	1.287	0.087
ascites	1.279	0.764	3.249	0.126
thrombosis	1.878	0.912	3.104	0.201
IPN	3.174	1.783	11.902	0.002

*APACHE* Acute Physiology and Chronic Health Evaluation, BMI body mass index, *EEN* the early enteral nutrition, *CRP* C-reactive protein, *IAP* intra-abdominal pressure, *MCTSI* modified CT severity index, *IPN* infected pancreatic and extra-pancreatic necrosis

**Table 3:** Independent risk factors in a multivariate logistic regression analysis of GIF

Variable	OR	95% CI		*P* value
		Lower	Upper	
Occurrence of IPN	3.012	1.693	15.026	0.004
EEN	0.267	0.182	0.738	0.016
MCTSI	1.183	1.096	2.547	0.037
B blood type	1.006	0.748	3.562	0.048

*IPN* infected pancreatic and extra-pancreatic necrosis, *EEN* the early enteral nutrition, *MCTSI* modified CT severity index

**Table 4:** General characteristics data of SAP with GIF

GIF style	Phase			Management			Death
	APFC	INP	WON	operation	drainage	Selfhealing	
Localization	2	12	26	0	32	8	7/40
diffusion	2	10	0	10	2	0	7/12
total	4	22	26	10	34	8	14/52

*APFC* acute pancreatic or peripancreatic fluid collection, *WON* walled-off necrosis, *IPN* infected pancreatic or peripancreatic necrosis

## Discussion

GIF is a well-recognized complication that occurs in the late phase of AP. However, the clinical relevance of GIF in patients with AP has been rarely studied by investigators, and the reported incidence ranges from 3 to 12% in different studies [28–30]. In the present retrospective study, GIF developed in 52 of 344 patients (15.12%), which was relatively higher than previously reported. The higher incidence should be mainly due to screening only SAP patients for enrollment in our study, and in addition, some critical patients admitted to our center who were transferred from other facilities.

We evaluated 15 potential risk factors for GIF in SAP patients and demonstrated the occurrence of IPN resulting from ANC or WON and high MCTSI to be independent risk factors (*P* = 0.004, OR = 3.012; *P* = 0.037, OR = 1.183). EEN acted as a protective factor for GIF with SAP (*P* = 0.0001, OR = 1.006). Unfortunately, our data suggested that blood type B was also correlated with GIF (*P* = 0.048, OR = 1.006), not only less strongly, but the 95% confidence interval (0.748^_^3.562) was paradoxical based on multivariate logistic regression.

Previous studies have confirmed infection of pancreatic necrosis can be observed in 25^_^70% of patients with necrotizing disease [31]. Occurrence of pancreatic and peripancreatic necrosis and formation of WON serve as nidus for bacterial superinfection are prone to develop infections which thought to be involved in the pathogenesis of GIF. The microbial pathogens that cause IPN in necrotizing pancreatitis are predominantly gut-derived [32]. A transition from a pro-inflammatory to an anti-inflammatory response occurs within the first 1^_^2 weeks, the patient is at risk for the translocation of intestinal flora as a result of intestinal barrier failure followed by the development of consequent IPN and fluid collections, which is thought to be associated with severe local inflammatory response and may erode the blood vessel directly, stimulate vessel spasm, enhance thrombosis, and reduce capillary perfusion, especially, when secondary infection occurs [33]. Inflammation or infected necrosis and enzyme-rich fluid can exacerbate the condition of gastrointestinal (GI) tract, which facilitate the formation of oedema, thrombosis, ischemia, necrosis and resulting in formation of fistula eventually [11]. With respect to the time of occurrence of GIF during the course of SAP, 85% patients had GIF beyond 4^_^8 weeks [34], which suggests that the development of GIF is associated with the long-term effects of the pancreatic or peripancreatic inflammation and infection. The finding is in agreement with our results, as patients with IPN had a higher risk of GIF. Hence, due to the anatomical characteristics of GI tract and the nature of pancreatic necrosis, the region of GIF was local or diffuse, but the underlying pathogenesis of both were same. Timely drainage of infected necrotizing collection could significantly decrease the risk of GIF.

For preventing infections in patients with SAP, recent studies have universally supported the optimal strategy of fluid resuscitation, which involves aggressive fluid administration during the first 24h of admission, highlight optimal targeting of individualized fluid requirements, and utilizing lactated Ringer’s as the fluid type of preferred choice [35–37]. Additionally, routine antibiotic or probiotic prophylaxis is recommended for patients with SAP. Antibiotic therapy should be initiated while the source of the infection is suspected or investigated [38].

Reliable evidence from several randomized controlled trials and meta-analyses comparing the outcomes of EN to parenteral nutrition (PN) in patients with AP has clearly shown the superiority of EN in decreasing the infectious complication rate, MOF, mortality, and length of hospitalization [39]. Our data suggest that EEN, in contrast to the maximum IPN and maximum WON level, acts as a protective factor for GIF secondary to SAP (*P* = 0.016, OR = 0.267). EN starting in the early phase (48^_^72h after admission) of SAP is superior to later EN (72 h after admission) and PN. Some studies have demonstrated that EEN can timely deliver nutritional support, while it preserves gut mucosal integrity, inhibits bacterial overgrowth and translocation, supports splanchnic metabolism, and mitigates the systemic inflammation and risk of infection [40, 41]. The results of a well-designed multicentric randomized clinical trial did not show positive effects of EEN (within 24 h after admission) against on-demand nutrition (48 h since admission), with the incidence of IPN as an endpoint. Conversely, feeding within the first 24 h might act as a burden, which might be of no benefit to prevent gut-derived infectious complications. Accordingly, it is not recommended to initiate feeding within first 24 h, rather feeding initiated 48 h after admission is more beneficial [42, 43]. However, SAP is always accompanied with delayed gastric emptying and intestinal ileus that lead to anorexia, nausea, and vomiting that prevent the patient from tolerating oral fluids and diet. And ventilator support executing sedation in the ICU preclude oral feeding in patients with SAP. So EN need to be supplied via nasogastric (NG), nasoduodenal, or nasojejunal (NJ) feeding. In patients who have gastric outlet obstruction from pancreatic inflammation or fluid collection related duodenal compression, a nasogastrojejunal (NGJ) tubing system, a double lumen tube with proximal gastric decompression, and distal jejunal feeding ports can be used to meet both the purposes without the need for two separate tubes [44, 45]. In our center, nasoduodenal feeding tubes as the primary method of enteral feeding were placed by endoscopists or radiologists, NJ and NGJ were managed secondarily if necessary, and fluid feeding was initiated after 48^_^120 h after admission.

It was confirmed in the present study that patients with SAP and high MCTSI scores were at a higher risk for GIF. The MCTSI is one of the most preferred modality for severity assessment of acute pancreatitis by incorporating extra-pancreatic complications. CECT is considered the non-invasive reference standard for diagnosing AP, and is highly accurate in assessing IPN and its complications when performed 72–96 h after symptom onset [24, 46]. MCTSI is credited with IPN and involvement of pleural effusion, ascites, vascular or gastrointestinal complications, and as expected, has the greatest accuracy for predicting SAP, which correlates more closely with patient outcome in terms of duration of hospital stay and development of organ failure [47, 48]. Because MCTSI is intrinsically implicated with gastrointestinal complications, which involves the potential opportunity for occurrence of GIF. MCTSI is inevitably as a sensitive risk factor for GIF in patients with SAP.

Previous reports have suggested that blood type B may be a genetic eliciting factor for chronic autoimmune pancreatitis [49]. Unfortunately, our results revealed a slight correlation between blood type B and the development of GIF in patients with SAP. For blood type B (*P* = 0.048,OR = 1.006), marginal statistical significance was reached, but there was an ambiguity with respect to the paradoxical 95% confidence interval (0.748^_^3.562) observed in the multivariate logistic regression, which indicated a weaker association of risk factor for GIF or implied a relatively limited sample size in our study. How the intrinsic relationship between these observed factors is confusedly unclear. Even though our data have shown an association between the development of GIF in patients with SAP and blood type B, previous analyses should not be regarded as an outcome of our study, and further investigation is warranted.

The clinical outcomes, as illustrated in Table 4 suggest that patients with SAP in combination with diffuse GIF have much longer hospital stays, more severe complications, extremely poor prognosis, and require more invasive treatments than localized counterparts, which might cause none of additional symptoms and are even detected incidentally [4, 50]. In our study, only 12 of 52 fistulas (23.1%) were shown to be diffuse GIF, and 40 of 52 fistulas (76.9%) were in localized GIF.

It was easy to neglect GIF because its symptom always overlap with clinical manifestations of SAP, and visible air pockets within the necrotic area on the imaging of CECT are frequently confused with infection of necrosis [51, 52]. Even diffuse GIF might not be observed timely unless persistent deterioration aroused attention. Nevertheless, the morbidity associated with localized GIF is significantly higher than diffuse GIF. Thus, it is not difficult to explain why there is no remarkably discrepancy in mortality (26.92% vs. 21.23%; *P* = 0.754) related to the SAP between patients with and without GIF, as shown in Table 1. This consistency may be mainly attributed to the following: First, the occurrence of severe intestinal edema, ischemia, necrosis and fistula caused by erosion and necrosis of enzyme-rich fluid and infected necrotic tissue is most localized in the retroperitoneal space and diffusion is limited. Therefore, most GIF of upper GI tract can usually close spontaneously with time if the infected source can be well controlled [12, 14]. Second, GIF can potentially benefit the patient by draining IPN into GI tract, especially when IPN, WON, or pseudocyst communicate with the gut [4, 53]. Third, advances in technology, a sufficient nutrition supply, effective anti-infective treatment, and timely surgical intervention have also played extremely important roles [54].

There were two primary advantages in the present study. First, we observed originally that blood type B is an independent risk factor for GIF in patients with SAP, albeit the relatively small sample of the present study might have reduced the statistical power. Second, we attempted to identify specific, routinely tested and reproducible baseline clinical parameters that predict the risk factors which are associated with GIF secondary to SAP. There were also several limitations to our study. First, this was a retrospective study with a relatively limited sample size. The actual incidence of GIF might be lower than our report because some patients transferred to our center were critical and most had been treated in other facilities for a long time. The non-parametric test applied may bring some uncertainty to the conclusions. Second, the guidelines that some experts recommended (the prophylactic administration of antibiotics and some pharmacologic agents are not necessary in all patients with acute pancreatitis) [55–58] were also not implemented in the present study owing to lacking of uniform experimental assessments. Third, as a relative contra-indication of EN, SAP was associated with delayed gastric emptying and intestinal ileus. EEN might be executed unsuccessfully due to subjective bias of the individual administrator. Finally, by introducing the updated classification of SAP, it was inevitable to make selection bias by ruling in or out patients who were over-or-underestimated due to the seemingly homologous definition. Whether or not these aspects could affect the incidence and analysis of GIF in patients with SAP is uncertain.

## Conclusion

We conclude that occurrence of IPN and higher MCTSI are independent significant risk factors for the development of GIF in patients with SAP. EN in 48–72 h after admission is conformed to be an independent significant protective factor of GIF secondary to SAP. Also the patient with blood type B is predisposed to develop GIF in the patient with SAP, perhaps which need more support.

## Abbreviations

ANC: Acute necrotic collection
APFC: Acute pancreatic or peripancreatic fluid collection
EEN: Early enteral nutrition
GIF: Gastrointestinal fistula
IPN: Defined when the presence of the ANCs and WONs development of infection
MCTSI: Modified CT severity index
SAP: Severe acute pancreatitis
WON: and walled-off necrosis

## References

[1] Petrov MS, Shanbhag S, Chakraborty M, Phillips AR, Windsor JA (2010). Organ failure and infection of pancreatic necrosis as determinants of mortality in patients with acute pancreatitis. Gastroenterology.

[2] Buter A, Imrie CW, Carter CR, Evans S, McKay CJ (2002). Dynamic nature of early organ dysfunction determines outcome in acute pancreatitis. Br J Surg.

[3] Petrov MS (2011). Predicting the severity of acute pancreatitis: choose the right horse before hitching the cart. Dig Dis Sci.

[4] Kochhar R, Jain K, Gupta V (2012). Fistulization in the GI tract in acute pancreatitis. Gastrointest Endosc.

[5] Talukdar R, Nechutova H, Clemens M, Vege SS (2013). Could rising BUN predict the future development of infected pancreatic necrosis?. Pancreatology.

[6] van Baal MC, van Santvoort HC, Bollen TL, Bakker OJ, Besselink MG, Gooszen HG (2011). Systematic review of percutaneous catheter drainage as primary treatment for necrotizing pancreatitis. Br J Surg.

[7] Flati G, Andrén-Sandberg A, La Pinta M, Porowska B, Carboni M (2003). Potentially fatal bleeding in acute pancreatitis: pathophysiology, prevention, and treatment. Pancreas.

[8] Beger HG, Rau BM (2007). Severe acute pancreatitis: Clinical course and management. World J Gastroenterol.

[9] Pezzilli R, Zerbi A, Campra D, Capurso G, Golfieri R, Arcidiacono PG (2015). Consensus guidelines on severe acute pancreatitis. Dig Liver Dis.

[10] Doberneck RC (1989). Intestinal fistula complicating necrotizing pancreatitis. Am J Surg.

[11] Tsiotos GG, Smith CD, Sarr MG (1995). Incidence and management of pancreatic and enteric fistulas after surgical management of severe necrotizing pancreatitis. Arch Surg.

[12] Mohamed SR, Siriwardena AK (2008). Understanding the colonic complications of pancreatitis. Pancreatology.

[13] Suzuki A, Suzuki S, Sakaguchi T (2008). Colonic fistula associated with severe acute pancreatitis: report of two cases. Surg Today.

[14] Van Minnen LP, Besselink MG, Bosscha K, van Leeuwen MS, Schipper ME, Gooszen HG (2004). Colonic involvement in acute pancreatitis. A retrospective study of 16 patients. Dig Surg.

[15] Yeom HJ, Yi SY (2007). Spontaneous resolution of pancreatic gastric fistula. Dig Dis Sci.

[16] Sun B, Li HL, Gao Y, Xu J, Jiang HC (2003). Factors predisposing to severe acute pancreatitis: evaluation and prevention. World J Gastroenterol.

[17] Sun B, Li HL, Gao Y, Xu J, Jiang HC (2003). Analysis and prevention of factors predisposing to infections associated with severe acute pancreatitis. Hepatobiliary Pancreat Dis Int.

[18] Banks PA, Bolen TL, Dervenis C, Gooszen HG, Johnson CD, Sarr MG (2013). Classifi cation of acute pancreatitis-2102: revision of the Atlanta classifi cation and defi nitions by international consensus. Gut.

[19] Bollen TL (2012). Imaging of acute pancreatitis: update of the revised Atlanta classifi cation. Radiol Clin North Am.

[20] Thoeni RF (2012). The revised Atlanta classifi cation of acute pancreatitis: its importance for the radiologist and its effect on treatment. Radiology.

[21] Banks PA, Freeman ML (2006). Practice parameters committee of the American college of gastroenterology. Practice guidelines in acute pancreatitis. Am J Gastroenterol.

[22] Papachristou GI, Muddana V, Yadav D, O’Connell M, Sanders MK, Slivka A (2010). Comparison of BISAP, Ranson’s, APACHE-II, and CTSI scores in predicting organ failure, complications, and mortality in acute pancreatitis. Am J Gastroenterol.

[23] Marshall JC, Cook DJ, Christou NV, Bernard GR, Sprung CL, Sibbald WJ (1995). Multiple organ dysfunction score: a reliable descriptor of a complex clinical outcome. Crit Care Med.

[24] Balthazar EJ, Robinson DL, Megibow AJ, Ranson JH (1990). Acute pancreatitis: value of CT in establishing prognosis. Radiology.

[25] Lenhart DK, Balthazar EJ (2008). MDCT of acute mild (nonnecrotizing pancreatitis): abdominal complications and fate of fl uid collections. Am J Roentgenol.

[26] Pelaez-Luna M, Vege SS, Petersen BT, Chari ST, Clain JE, Levy MJ (2008). Disconnected pancreatic duct syndrome in severe acute pancreatitis: clinical and imaging characteristics and outcomes in a cohort of 31 cases. Gastrointest Endosc.

[27] Salomone T, Tosi P, Palareti G (2003). Coagulative disorders in human acute pancreatitis: role for the D-dimer. Pancreas.

[28] Falconi M, Pederzoli P (2001). The relevance of gastrointestinal fistulae in clinical practice: a review. Gut.

[29] Forsmark CE (2001). The clinical problem of biliary acute necrotizing pancreatitis: epidemiology, pathophysiology, and diagnosis of biliary necrotizing pancreatitis. J Gastrointest Surg.

[30] Urakami A, Tsunoda T, Hayashi J (2002). Spontaneous fistulization of a pancreatic pseudocyst into the colon and duodenum. Gastrointest Endosc.

[31] Singh RK, Poddar B, Baronia AK, Azim A, Gurjar M, Singhal S (2012). Audit of patients with severe acute pancreatitis admitted to an intensive care unit. Indian J Gastroenterol.

[32] Besselink MG, van Santvoort HC, Boermeester MA (2009). Timing and impact of infections in acute pancreatitis. Br J Surg.

[33] Noor MT, Radhakrishna Y, Kocchar R, Kocchar R, Wig JD, Sinha SK (2011). Bacteriology of infection in severe acute pancreatitis. JOP.

[34] Dellinger EP, Tollado JM, Soto NE, Ashley SW, Barie PS, Dugernier T (2007). Early antibiotic treatment for severe acute necrotizing pancreatitis: a randomized, double-blind, placebo-controlled study. Ann Surg.

[35] Working Group IAP/APA Acute Pancreatitis Guidelines (2013). IAP/APA evidence-based guidelines for the management of acute pancreatitis. Pancreatology.

[36] Hartman H, Sippola T, Kupcinskas J, Lindström O, Johnson C, Regnér S (2013). Raised intestinal fatty acid binding protein correlates to severe acute pancreatitis. Abstract presented at the 45th annual meeting of the European Pancreatic Club, June 26–29, 2013, Zurich, Switzerland. Pancreatology.

[37] Rahman SH, Ammori BJ, Holmfi eld J, Larvin M, McMahon MJ (2003). Intestinal hypoperfusion contributes to gut barrier failure in severe acute pancreatitis. J Gastrointest Surg.

[38] Pezzilli R (2009). Pharmacotherapy for acute pancreatitis. Expert Opin Pharmacother.

[39] Singh N, Sharma B, Sharma M, Sachdev V, Bhardwaj P, Mani K, Joshi YK, Saraya A (2012). Evaluation of early enteral feeding through nasogastric and nasojejunal tube in severe acute pancreatitis: a noninferiority randomized controlled trial. Pancreas.

[40] Marik PE, Zaloga GP (2004). Meta-analysis of parenteral nutrition versus enteral nutrition in patients with acute pancreatitis. BMJ.

[41] Al-Omran M, Albalawi ZH, Tashkandi MF, Al-Ansary LA (2010). Enteral versus parenteral nutrition for acute pancreatitis. Cochrane Database Syst Rev.

[42] Petrov MS, van Santvoort HC, Besselink MG, van der Heijden GJ, Windsor JA, Gooszen HG (2008). Enteral nutrition and the risk of mortality and infectious complications in patients with severe acute pancreatitis:a meta-analysis of randomized trials. Arch Surg.

[43] McClave SA, Heyland DK (2009). The physiologic response and associated clinical benefi ts from provision of early enteral nutrition. Nutr Clin Pract.

[44] O’Keefe S, Rolniak S, Raina A, Graham T, Hegazi R, Centa-Wagner P (2012). Enteral feeding patients with gastric outlet obstruction. Nutr Clin Pract.

[45] Kumar A, Singh N, Prakash S, Saraya A, Joshi YK (2006). Early enteral nutrition in severe acute pancreatitis: a prospective randomized controlled trial comparing nasojejunal and nasogastric routes. J Clin Gastroenterol.

[46] De Waele JJ, Delrue L, Hoste EA, De Vos M, Duyck P, Colardyn FA (2007). Extrapancreatic infl ammation on abdominal computed tomography as an early predictor of disease severity in acute pancreatitis: evaluation of a new scoring system. Pancreas.

[47] Bollen TL, Singh VK, Maurer R, Repas K, van Es HW, Banks PA (2012). A comparative evaluation of radiologic and clinical scoring systems in the early prediction of severity in acute pancreatitis. Am J Gastroenterol.

[48] Mortele KJ, Wiesner W, Intriere L, Shankar S, Zou KH, Kalantari BN (2004). A modifi ed CT severity index for evaluating acute pancreatitis: improved correlation with patient outcome. AJR Am J Roentgenol.

[49] Weiss FU, Schurmann C, Teumer A, Mayerle J, Simon P, Völzke H, Greinacher A, Kuehn JP, Zenker M, Völker U (2016). ABO blood type B and fucosyltransferase 2 non-secretor status as genetic risk factors for chronic pancreatitis. Gut.

[50] Gardner A, Gardner G, Feller E (2003). Severe colonic complications of pancreatic disease. J Clin Gastroenterol.

[51] Van Baal MC, Bollen TL, Bakker OJ, van Goor H, Boermeester MA, Dejong CH (2014). The role of routine fi ne-needle aspiration in the diagnosis of infect ed necrotizing pancreatitis. Surgery.

[52] Balthazar EJ (2002). Complications of acute pancreatitis: clinical and CT evaluation. Radiol Clin North Am.

[53] Levy I, Ariche A (1999). Complete recovery after spontaneous drainage of pancreatic abscess into the stomach. Scand J Gastroenterol.

[54] van Santvoort HC, Bakker OJ, Bollen TL, Besselink MG, Ahmen Ali U, Schrijver AM (2011). A conservative and minimally invasive approach to necrotizing pancreatitis improves outcome. Gastroenterology.

[55] Delcenserie R, Yzet T, Ducroix JP (1996). Prophylactic antibiotics in treatment of severe acute alcoholic pancreatitis. Pancreas.

[56] Sharma VK, Howden CW (2001). Prophylactic antibiotic administration reduces sepsis and mortality in acute necrotizing pancreatitis: a meta-analysis. Pancreas.

[57] Takeda K, Yamauchi J, Shibuya K, Sunamura M, Mikami Y, Matsuno S (2001). Benefit of continuous regional arterial infusion of protease inhibitor and antibiotic in the management of acute necrotizing pancreatitis. Pancreatology.

[58] Griesbacher T (2000). Kallikrein-kinin system in acute pancreatitis: potential of B(2)-bradykinin antagonists and kallikrein inhibitors. Pharmacology.

